# An Unusual Presentation of Systemic Lupus Erythematosus as Hemophagocytic Lymphohistiocytosis in a Male.

**DOI:** 10.7759/cureus.5427

**Published:** 2019-08-19

**Authors:** Mathew Thomas, Alex Robert, Neenu Kuruvilla, Uthamanand C

**Affiliations:** 1 Breast Medical Oncology, Cleveland Clinic, Cleveland, USA; 2 Internal Medicine, Church of South India (CSI) Holdsworth Memorial Hospital, Mysore, IND; 3 Internal Medicine, Government Medical College Kottayam, Kottayam, IND

**Keywords:** hlh, male sle, autoimmune, rare presentation

## Abstract

Hemophagocytic lymphohistiocytosis (HLH) is an aggressive and life-threatening hyper-inflammatory condition characterized by excessive activation of macrophages and T cells resulting in multi-organ dysfunction. HLH can be primary/familial or secondary to infections, malignancy, immunosuppression, and autoimmune conditions. Systemic lupus erythematosus (SLE) is an autoimmune condition that can predispose to HLH. SLE, as other immune conditions is more common in females than in males. However, the occurrence of SLE in males and subsequent predisposition to HLH is rare. We report the case of a 22-year-old gentleman who presented with fever for three months and one day of altered sensorium prior to admission. On evaluation, he fulfilled five out of the 17 diagnostic criteria for SLE. His bone marrow biopsy showed hemophagocytosis and met five out of the eight diagnostic criteria of HLH, and a diagnosis of HLH secondary to SLE was made. He was treated with pulse doses of intravenous methylprednisolone and azathioprine and showed dramatic improvement. A high index of suspicion is essential for the diagnosis of HLH and prompt initiation of treatment is of utmost importance for tackling such a rapidly progressive life-threatening condition.

## Introduction

Hemophagocytic lymphohistiocytosis (HLH) is an aggressive and potentially fatal condition characterized by immune activation leading to multi-organ dysfunction. HLH can be inherited in an autosomal recessive fashion, but can also be secondary to infections, malignancy, immunosuppression and autoimmune conditions [1]. Primary HLH is more common in the pediatric population, while secondary HLH is more common among adults [1]. HLH involves inappropriate activation of T cells and macrophages, which produces pro-inflammatory cytokines [1]. Common manifestations of HLH include prolonged fever, hepatosplenomegaly, pancytopenia, and elevated levels of liver enzymes, triglyceride, and ferritin. Systemic lupus erythematosus (SLE) is an autoimmune condition that can predispose to HLH. The occurrence of SLE in males and subsequent predisposition to HLH is rare, with a prevalence of 0.9% to 4.6 % [2]. Here, we report a rare case of young male with SLE, whose initial manifestation was HLH.

## Case presentation

A 22-year-old male, hotel employee by profession, with no significant past medical history and family history, presented with fever for three months and one day of altered sensorium. He had intermittent low grade fever for three months, but it got worsened over the five days prior to admission. It was associated with chills and with 1-2 episodes/day of vomiting for three days. He also had a history of leg swelling, facial puffiness, and abdominal distension for three days. There was no history of chest pain, shortness of breath, palpitations, headache, syncope, or seizures. He denied any history of smoking, excessive alcohol use, or substance abuse. On examination, the patient was drowsy and disoriented with a Glasgow Coma Score (GCS) of 12/15, temperature of 101 °F, pulse rate of 96/min, blood pressure of 132/84 mm Hg, and Spo2 of 96% in room air. Physical examination showed the presence of pallor, facial puffiness, and bilateral pitting pedal edema. Systemic examination showed the presence of hepatosplenomegaly and shifting dullness. There were no signs of meningeal irritation, no focal deficits, and optic fundus examination was normal.

Investigations and treatment

Laboratory results at presentation (Table 1) were significant for pancytopenia, hyponatremia, hypoalbuminemia, hyperbilirubinemia, and elevated liver enzymes.

**Table 1:** Laboratory results at presentation MCV:* *mean corpuscular volume; ESR: erythrocyte sedimentation rate; AST: aspartate aminotransferase; ALT: alanine aminotransferase; ALP: alkaline phosphatase; PT: prothrombin time; INR: international normalized ratio; MP-QBC: malarial parasite-quantitative buffy coat; RBC: red blood cell; hpf: high power field

Variable	Measurement	Reference values
Hemoglobin (g/dL)	5.8	13.5-17.5
Total leucocyte count (TLC) (/mm3)	1000	4,500-11,000
Neutrophil (%)	79	54-62
Lymphocytes (%)	12	25-33
Monocytes (%)	4	3-7
Eosinophil (%)	0.8	1-3
Basophil (%)	2.4	0-0.75
Platelet count(/mm3)	66000	150,000-400,000
MCV (μm3)	75	80-100
ESR (mm/h)	18	0-15
Sodium (mEq/L)	128	136-145
Potassium (mEq/L)	5.2	3.5-5.0
Chloride (mEq/L)	99	95-105
Blood urea nitrogen (mmol/dL)	98	8-24
Creatinine (mg/dL)	1.1	0.6-1.2
Total protein (g/dL)	4.8	6.0-7.8
Albumin (g/dL)	1.7	3.5-5.5
Total bilirubin (mg/dL)	1.4	0.1-1.0
Direct bilirubin (mg/dL)	1.0	0.0-0.3
AST (U/L)	159	8-40
ALT (U/L)	34	8-40
ALP (U/L)	214	30-100
PT (seconds)	13.6	11-15
INR	0.71	0.8-1.2
MP-QBC	Negative	
Urine-albumin, sugar	Nil	
Urine-pus cells	1-2/hpf	
Urine-RBC	Nil	

Computed tomography (CT) of the brain was normal. Ultrasound of abdomen and pelvis showed hepatosplenomegaly with moderate-to-severe ascites. Results of other investigations including peripheral smear, infectious disease panel, and Coombs test are shown in Table 2. The initial differential diagnoses were autoimmune, infectious, or inflammatory conditions. So he was started on empiric doxycycline, meropenem, hydrocortisone, fluconazole, and other supportive measures.

**Table 2:** Infectious disease panel ELISA: enzyme-linked immunosorbent assay; RBC: red blood cell; HBsAg: hepatitis B surface antigen; HCV: hepatitis C virus; IgM: immunoglobulin M; HIV: human immunodeficiency virus; TSH: thyroid-stimulating hormone

Variable	Measurement	Reference values
Peripheral smear	Microcytic hypochromic RBC, leucopenia, thrombocytopenia, no blasts	
HIV ELISA	Negative	
HBsAg	Negative	
Anti-HCV antibody	Negative	
Reticulocyte Count	1%	0.5%-1.5% of red cells
Weil-Felix test	Negative	
Widal test	Negative	
IgM Scrub typhus	Negative	
IgM Brucella	Negative	
IgM Leptospira	Negative	
IgM Dengue	Negative	
TSH (μU/mL)	0.63	0.5-5.0
Direct Coombs test	Negative	
Indirect Coombs test	Negative	
Blood Culture	No growth (48 hrs) and after 5 days	

**Figure 1:**
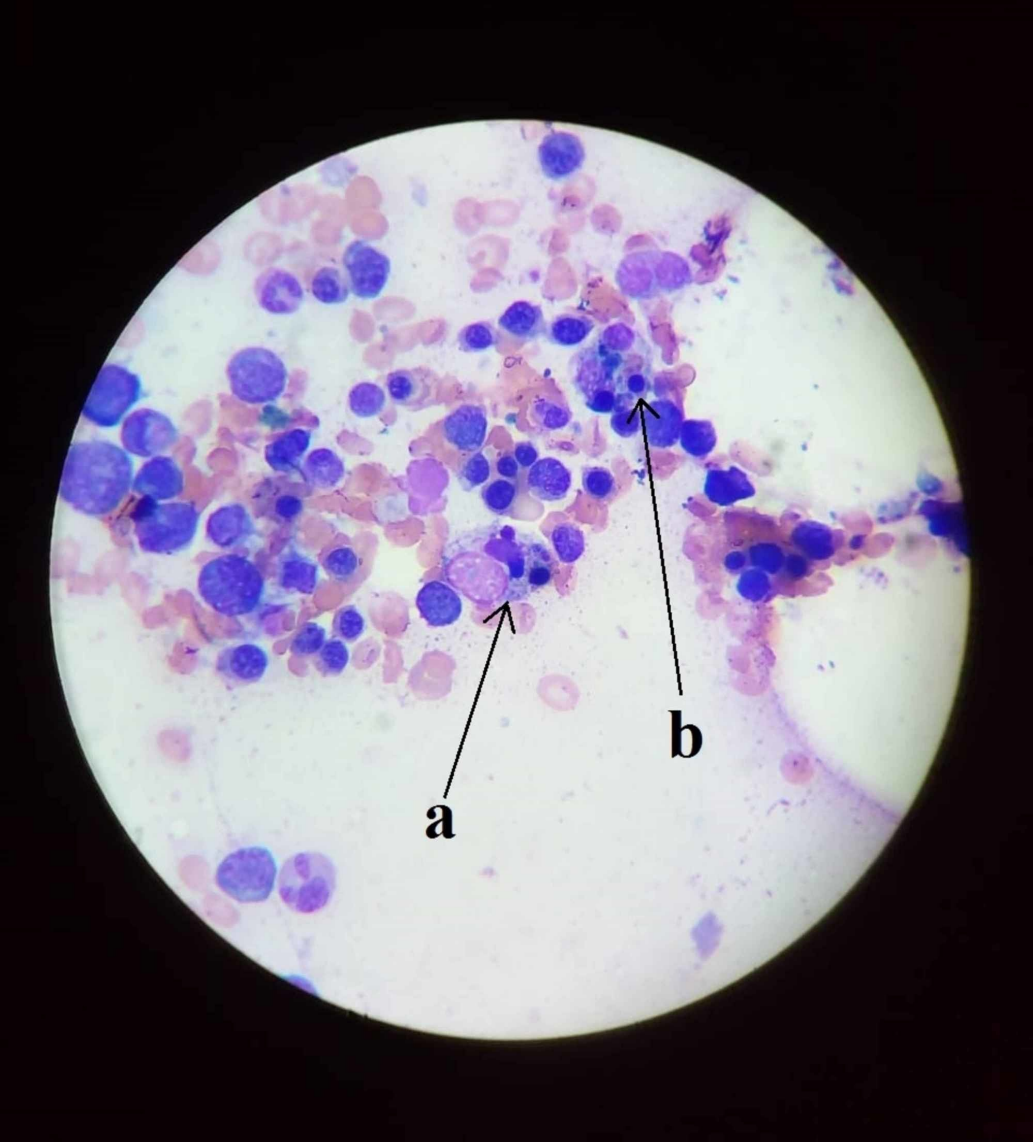
Bone marrow biopsy showing hemophagocytosis and lupus erythematosus cell phenomenon (a) Lupus erythematosus cell, (b) phagocytosis by histiocyte

Then, he underwent bone marrow biopsy and the smear showed histiocytes with erythrophagocytosis and engulfment of lymphocytes, and also the presence of lupus erythematosus (LE) cells (neutrophil or macrophage that has phagocytosed the nuclear material of another cell) phenomenon (Figure 1). Hence, he was evaluated for SLE and blood levels of ferritin and lactate dehydrogenase (LDH) and revealed elevated levels of ferritin and LDH, and also high titers of antinuclear antibody (ANA) and positive antidouble stranded DNA (anti-dsDNA) (Table 3).The patient met the diagnostic criteria for SLE and HLH, and a diagnosis of HLH secondary to SLE was made.

**Table 3:** Autoimmune and hemophagocytic lymphohistiocytosis panel LDH: lactate dehydrogenase; ANA: antinuclear antibody; Anti-ds DNA: antidouble stranded DNA

Variable	Measurement	Reference values
LDH	2944	45-90 U/L (100-250 IU/L)
Ferritin (ng/mL)	1896	15-200
ANA		Impression
Intensity	3+	Positive ANA if there is positive reaction with 1:100 titer
Titer	1:100	
Pattern	Nuclear homogenous	
Impression	positive	
Anti-dsDNA	positive	

He was then treated with pulse dose steroids with intravenous methylprednisolone 1000 mg daily for five days and then switched to prednisolone 60 mg orally (PO) once daily. With blood transfusions and other supportive measures, his blood counts improved. Later, steroids were tapered and azathioprine 50 mg PO twice daily was added. His general condition improved over the next one week. There were no more episodes of fever and altered sensorium, and he was discharged after three weeks on steroids and immunosuppressants. Follow-up visit at one week showed significant improvement. Now he is on regular outpatient follow up and is on azathioprine PO daily and tapering doses of steroids. He is doing well clinically and back to his routine day to day work.

## Discussion

We report a case of young male patient whose SLE manifested as HLH. His clinical and laboratory findings indicated pancytopenia, positive ANA and anti-ds DNA antibody, meeting five out of 17 (three clinical and two immunological) of the Systemic Lupus International Collaborating Clinics (SLICC) criteria for the diagnosis of SLE [3]. He had fever, pancytopenia, splenomegaly, bone marrow hemophagocytosis, and hyperferritinemia, fulfilling five out of the eight diagnostic criteria of HLH, described in the HLH 2004 trial [4-5] . Hence, the patient was diagnosed with SLE manifesting as HLH.

HLH can be primary resulting from a genetic defect leading to decreased cytotoxic activity of Natural Killer (NK) cells and cytotoxic T cells [2]. Secondary HLH results from triggering agents like infections or malignancies but can also be induced by autoimmune conditions, in which case it is called macrophage activation syndrome (MAS) [1]. Inability to clear the antigens secondary to these conditions lead to hyperactivation of the immune system, progressing to cytokine storm, and results in organ dysfunction [1]. HLH possesses diagnostic challenges as it has overlapping features with sepsis and multiple organ dysfunction syndrome (MODS) [1].

The treatment of HLH is based on the HLH-94 protocol. Induction/initial therapy consists of etoposide (twice weekly during the first two weeks, and then weekly), in combination with dexamethasone [6]. Central nervous system (CNS) disease is treated with intrathecal methotrexate. For patients with resistant or relapsing disease, continuation therapy with pulse doses of dexamethasone in combination with etoposide and cyclosporine is recommended to keep them in remission until allogeneic hematopoietic stem cell transplant could be performed.

In 2004, the HLH-2004 protocol was initiated (NCT00426101). It differs from HLH-94 protocol by using cyclosporine from day one of the initial treatment phase, and the addition of intrathecal steroids to intrathecal methotrexate for CNS disease. The HLH-94 and 2004 protocols are pediatric protocols, and may result in overtreatment and toxic when used in adults. Hence, dose reductions and individualized approach should be considered [1].

Antithymocyte globulin is an immunosuppressant effective in familial HLH [7]. Emapalumab is an anti-interferon (IFN)-γ monoclonal antibody approved by the Food and Drug Administration (FDA) in 2018, for the treatment of primary HLH in adults and children with refractory, recurrent, or progressive disease [8]. Alemtuzumab is an anti CD-52 monoclonal antibody, considered as an effective salvage treatment agent for refractory HLH, leading to improvement and survival benefit to hematopoietic stem cell transplant [9].

Treatment of secondary HLH is aimed at treating the underlying condition. In HLH due to SLE, high dose steroids and immunosuppressive agents including cyclosporine, cyclophosphamide, and intravenous immunoglobulin are effective [10]. Aoyama-Maeda et al. reported a case of MAS secondary to SLE, successfully treated with intravenous immunoglobulin (IVIG) and oral tacrolimus [11]. There are reported cases of treatment refractory SLE induced HLH, managed successfully with rituximab and infliximab [10, 12]. Our patient was treated with azathioprine and high dose steroids, and showed significant clinical improvement.

Mortality rate in adult HLH is high, ranging from 20% to 88%, mainly due to secondary infections, refractory HLH and progression of the underlying secondary causes [1].

The co-occurrence of SLE and HLH have overlapping clinical features and hence high level of suspicion is necessary for diagnosis. Prompt initiation of treatment for HLH is of extreme importance as it is a rapidly progressive life-threatening condition, even with adequate treatment.

## Conclusions

SLE in males and SLE presenting as HLH is rare. HLH and SLE has overlapping clinical features and thus possess diagnostic challenges. Treatment of secondary HLH is aimed at treating the underlying cause. High-dose steroids and immunosuppressive agents are effective in SLE-induced HLH. Mortality rate of HLH is high and hence prompt initiation of treatment is of utmost importance.
